# Editorial: Sequencing and phylogenetic analysis as a tool in molecular epidemiology of veterinary infectious diseases, Volume II

**DOI:** 10.3389/fvets.2025.1726749

**Published:** 2025-11-06

**Authors:** Amro Hashish, Mohamed Maarouf, Iryna V. Goraichuk

**Affiliations:** 1 Department of Veterinary Diagnostic and Production Animal Medicine, College of Veterinary Medicine, Iowa State University, Ames, IA, United States; 2 National Laboratory for Veterinary Quality Control on Poultry Production, Animal Health Research Institute, Agriculture Research Center, Giza, Egypt; 3 Key Laboratory of Animal Pathogen Infection and Immunology of Fujian Province, College of Animal Sciences, Fujian Agriculture and Forestry University, Fuzhou, China; 4 Department of Virology, Faculty of Veterinary Medicine, Suez Canal University, Ismailia, Egypt; 5 Exotic and Emerging Avian Viral Disease Research Unit, Southeast Poultry Research Laboratory, U.S. National Poultry Research Center, Agricultural Research Service, United States Department of Agriculture, Athens, GA, United States

**Keywords:** whole-genome sequencing (WGS), phylogenetic analysis, bioinformatics, molecular epidemiology, next-generation sequencing (NGS)

Over the past decade, rapid advances in sequencing technologies have transformed molecular epidemiology from a confirmatory discipline into a proactive framework for real-time pathogen tracking and evolutionary insight. Volume II of this Research Topic builds upon the foundation of the first collection, emphasizing how the continuous improvement of sequencing technologies and bioinformatic analysis enables more accurate detection, classification, and monitoring of a broad spectrum of veterinary pathogens. The 14 contributions encompass poultry, swine, cattle, small ruminants, companion animals, and wildlife, demonstrating that genomics underpins outbreak investigation, routine surveillance, and discovery of hidden pathogen diversity. Together, they illustrate how high-throughput sequencing, long-read platforms, and integrative phylogenetic pipelines are now essential tools for modern disease control in animal populations.

## Avian and poultry pathogens

Several contributions focused on avian viruses of major economic and zoonotic importance. Si, Lee, et al. report the first detection of clade 2.3.4.4b H5N1 highly pathogenic avian influenza (HPAI) in a wild leopard cat in South Korea, where whole-genome sequencing (WGS) revealed mammalian-adaptive mutations and close phylogenetic relationships with contemporary avian H5N1 isolates, highlighting a cross-species spillover event detected only through genomics. This case exemplifies how sequencing enables rapid cross-species tracking and identification of molecular markers of host adaptation.

In wild waterfowl, Si, Kim, et al. also sequenced two H5N1 HPAI isolates collected in October 2024. Phylogenetic analysis showed each virus had a distinct origin: one belonged to genotype G2d and clustered with Japanese H5N1 viruses, while the other was genotype G2c and emerged from reassortment between clade 2.3.4.4b H5 and Eurasian low-pathogenic AIV genes. Together, these findings outline the continual reshuffling characteristic of avian influenza evolution and underscore the value of full-genome sequencing for tracing migratory-bird-mediated transmission routes.

In wild birds, Seo et al. investigated an outbreak of HPAI H5N1 clade 2.3.4.4b in hooded cranes (*Grus monacha*) in South Korea and demonstrated through WGS that the virus shared genetic continuity with Japanese strains, suggesting cross-border transmission through migratory flyways. The study emphasizes the necessity of coordinated international genomic surveillance of wild birds to monitor viral evolution.

In poultry, Zhu et al. analyzed the VP2 gene of 31 infectious bursal disease virus (IBDV) isolates from broiler flocks in southern China, uncovering co-circulation of very virulent, classical, and novel variant lineages. Notably, the novel variant isolates formed a distinct phylogenetic cluster, suggesting antigenic drift with potential vaccine implications. In Italy, Legnardi et al. developed and validated a lineage-specific RT-qPCR assay for the divergent infectious bronchitis virus (IBV) genotype GVIII and applied it to field samples, detecting GVIII-2 (IB80-like) viruses in 13.1% of flocks. These Italian isolates formed a tight phylogenetic clade with known GVIII-2 references, indicating that this divergent IBV lineage has been circulating undetected in the field, which underscores the need to update diagnostic assays and vaccination strategies.

Collectively, these investigations demonstrate how WGS, supported by standardized bioinformatic workflows, now form the backbone of avian-virus surveillance. They enable simultaneous detection of reassortment, emergence of divergent lineages, and transboundary dissemination, transforming avian-pathogen monitoring into a globally networked genomic enterprise.

## Swine pathogens

Genomic surveillance reveals highly dynamic viral populations in swine. Kang et al. conducted a molecular survey of porcine reproductive and respiratory syndrome virus (PRRSV) in China, sequencing 37 field isolates. They identified both PRRSV-1 and PRRSV-2 subtypes, including NADC30-like, VR-2332-like, novel recombinant, and a USA-like L1C.5 lineages, revealing complex genetic diversity circulating in regional herds. Chandra et al. analyzed over 114,000 U.S. PRRSV ORF5 sequences spanning 18 years, revealing lineage turnover and recombinant sub-clusters through automated clustering and Bayesian analysis. This large-scale approach exemplifies how sustained sequencing and open databases enable continuous, high-resolution monitoring of viral evolution. Comprehensive molecular monitoring and WGS provided the resolution needed to distinguish co-circulating genotypes and detect recombination events unseen by conventional assays.

Tamiozzo et al. used a two-locus MLVA scheme on *Mycoplasma hyopneumoniae* datasets from six countries, identifying 185 unique MLVA types. Bayesian clustering revealed three major global genotype clusters, with ~43% of types showing mixed ancestry, indicating a largely non-clonal, highly diverse population structure. Although fragment-based, this approach complements sequencing by mapping population structure where whole genomes remain limited.

Together, these studies illustrate how sequencing-based surveillance now operates from local genome recovery to national-level modeling. Integration of recombination-aware phylogenetics and automated analytics is transforming swine-health management into a continuously updated genomic ecosystem.

## Ruminant and other livestock viruses

Sequencing also advanced our understanding of ruminant viruses. Zhang P. et al. obtained the complete genome of an exogenous Jaagsiekte sheep retrovirus (JSRV) from Inner Mongolia and constructed a full-length infectious clone to study sheep pulmonary adenocarcinoma pathogenesis and retroviral transmission in flocks. In cattle, Hao et al. characterized a novel cytopathic bovine viral diarrhea virus BVDV-1d strain from Mongolia and showed that co-infection with non-cytopathic BVDV enhanced disease severity, insights obtainable only through molecular characterization and experimental validation. Yan et al. identified Aichivirus D (AiV-D) genotype D2 in Chinese dairy cattle, the first detection outside Japan, and generated its complete genome with unique substitutions suggesting adaptation. These investigations show how complete-genome and comparative-phylogenetic analyses link genetic variability to clinical outcomes, guiding vaccine and diagnostic refinement.

Expanding the scope from single-pathogen studies, Zhang S. et al. also performed a metaviromic survey of 688 sheep from northwestern China, revealing 38 virus families in nasal and anal swabs. Such untargeted sequencing uncovers the hidden virome of small ruminants and demonstrates how metagenomics identifies novel or low-abundance viruses without prior reference genomes. Phylogenetic analysis of assembled sequences revealed close evolutionary ties between the ovine viruses and those from other ruminants, suggesting cross-species circulation. Overall, whole-genome, clone-based, and metagenomic approaches now converge to deliver a comprehensive view of pathogen evolution and host adaptation, establishing a foundation for mechanistic molecular epidemiology in livestock research.

## Emerging viruses and one health

Several studies underscore the One Health interface between animal and human pathogens. Loy et al. performed extensive SARS-CoV-2 surveillance in Nebraska wildlife, zoo animals, and pets. White-tailed deer showed high seroprevalence, and recurrent Delta-variant detection was confirmed by genome sequencing, consistent with repeated human-to-deer spillover events. By contrast, zoo animals and domestic cats showed minimal infection. These findings highlight the value of broad genomic surveillance at the human–animal interface.

In companion animals, Sohn et al. used next-generation sequencing (NGS) to study an outbreak of systemic feline calicivirus (FCV) in South Korean pet cats, obtaining five complete genomes forming a novel Korean clade with virulence-associated mutations. A tiled-amplicon protocol enabled rapid assembly and detection of pathogenic mutations, illustrating sequencing's role in accelerating outbreak response in companion species.

Together, these studies demonstrate how genome-based surveillance bridges veterinary and public-health sectors, revealing zoonotic spillovers and host adaptation. Integration of animal-derived genomes into shared databases enhances One Health preparedness and early detection of emerging variants.

## Technological and methodological advances

The Research Topic also encompassed diverse genomic approaches. Most teams used Illumina short-read or Sanger sequencing for genome recovery or key gene targets. Legnardi et al. designed a new genotype-specific RT-qPCR for IBV GVIII surveillance, filling a gap in surveillance. Large-scale bioinformatics analyses were also employed: Chandra et al. compiled over 114,000 U.S. PRRSV ORF5 sequences to trace shifting viral lineages over 18 years. Tamiozzo et al. applied MLVA genotyping for *M. hyopneumoniae*, offering population insights where WGS is scarce. Together, these methods exemplify how combining traditional assays with sequencing and informatics enhances pathogen detection and interpretation.

Sequencing strategies ranged from tiled-amplicon Illumina and Nanopore protocols to metagenomics and hybrid assemblies. Integration of bioinformatic pipelines for recombination detection, lineage assignment, and molecular-clock analysis signals a field-wide move toward standardized, reproducible, and cloud-enabled genomic epidemiology. Adoption of FAIR-data principles strengthens global comparability and accessibility.

## Conclusions and future directions

Volume II of this Research Topic reinforces that sequencing and phylogenetic tools are indispensable in veterinary infectious disease research. The studies cover avian, swine, ruminant, companion-animal, and wildlife pathogens, unified by genomic analysis. Genomics-enabled surveillance reveals novel variants and cross-species transmission events often missed by routine diagnostics. Continued innovation in real-time sequencing, adaptive sampling, and AI-assisted epidemiological modeling promises further progress. Portable sequencers and cloud-based pipelines are making field genomics achievable, turning pathogen tracking into a continuous process. As costs decline and workflows standardize, sequencing will shift from retrospective analysis to predictive, proactive surveillance. Overall, this Research Topic captures a pivotal stage in veterinary molecular epidemiology: the transition from sequencing as a supplementary diagnostic tool to its role as the technological engine driving our understanding of pathogen evolution, transmission, and control.

